# [Ln_6_O_8_] Cluster‐Encapsulating Polyplumbites as New Polyoxometalate Members and Record Inorganic Anion‐Exchange Materials for ReO_4_^−^ Sequestration

**DOI:** 10.1002/advs.201900381

**Published:** 2019-06-17

**Authors:** Jian Lin, Lin Zhu, Zenghui Yue, Chuang Yang, Wei Liu, Thomas E. Albrecht‐Schmitt, Jian‐Qiang Wang, Shuao Wang

**Affiliations:** ^1^ Key Laboratory of Interfacial Physics and Technology Shanghai Institute of Applied Physics Chinese Academy of Sciences 2019 Jia Luo Road Shanghai 201800 P. R. China; ^2^ State Key Laboratory of Environmental‐Friendly Energy Materials School of National Defence Science & Technology and National Co‐Innovation Center for Nuclear Waste Disposal and Environmental Safety Southwest University of Science and Technology Sichuan Mianyang 621010 P. R. China; ^3^ State Key Laboratory of Radiation Medicine and Protection School for Radiological and Interdisciplinary Sciences (RAD‐X) and Collaborative Innovation Center of Radiation Medicine of Jiangsu Higher Education Institutions Soochow University Suzhou 215123 P. R. China; ^4^ Department of Chemistry and Biochemistry Florida State University 95 Chieftain Way Tallahassee FL 32306 USA

**Keywords:** clusters, ion exchange, perrhenate, plumbite, polyoxometalates

## Abstract

Various types of polyoxometalates (POMs) have been synthesized since the 19th century, but their assortment has been mostly limited to Groups 5 and 6 metals. Herein, a new family of POMs composed of a carbon group element as the addenda atoms with two distinct phases, **LnPbOClO_4_‐1** (Ln = Sm to Ho, Y) and **LnPbOClO_4_‐2** (Ln = Er and Tm) is reported. Both structures are built from [Ln_6_O_8_] rare‐earth metal hexamers being incorporated in [Pb_18_O_32_]/[Pb_12_O_24_] polyplumbites, and unbound perchlorates as charge‐balancing anions. Impressively, **YPbOClO_4_‐1** and **ErPbOClO_4_‐2** exhibit exceptional uptake capacities (434.7 and 427.7 mg g^−1^) toward ReO_4_^−^, a chemical surrogate for the key radioactive fission product in the nuclear fuel cycle ^99^TcO_4_^−^, which are the highest values among all inorganic anion‐exchange materials reported until now. The sorption mechanism is clearly elucidated and visualized by single‐crystal‐to‐single‐crystal structural transformation from **ErPbOClO_4_‐2** to a perrhenate‐containing complex **ErPbOReO_4_**, revealing a unique ReO_4_^−^ uptake selectivity driven by specific interaction within Pb···O‐ReO_3_^−^ bonds.

The chemistry of polyoxometalate (POM) has been extensively studied for decades, owning to their aesthetic structural diversity and broad functionality in areas of catalysis, photolytic reduction, medicine, etc.^1^ Numerous *iso*‐POM nanoaggregates have been reported but their building units are mostly limited to oxoanions based on the Groups 5 and 6 transition metals (Mo, W, V, Nb, and Ta).^2^ Those metals in their highest oxidation states are typically four‐ to seven‐coordinate and the metal cores are normally off‐centered due to the presence of short bonds between transition metals and yl O atoms. An emerging derivative of POMs is uranyl peroxide clusters, which are built from UO_2_^2+^ cations with two *trans* yl oxo groups, and more than 120 uranyl peroxide clusters featuring over 50 geometries have been reported.^3^ The tunable dimensions of POMs with a myriad of high number of metal centers per anion (up to 368) result in the encapsulation of heavy metal cations in the nanocages, which could not only retain the individual properties of POMs but also give rise to novel functions attributed from the encapsulated metal centers.^4^ Despite the abundance of POMs, polymolybdates and polytungstates are the dominant derivatives.^5^ Extending the species of POMs to other groups of elements and developing of new synthetic protocols for new family of POMs are of necessity.

Plumbite appears to be a logical candidate anion for hierarchical arrangement of moieties from monomeric anion to POMs for two reasons. First, Pb in plumbite is naturally off‐centered owning to the presence of stereochemically active lone‐pair electrons on the metal centers.^6^ As a result, the Pb−O−Pb bridges introduce a corrugated geometry when plumbites are properly aligned, providing the curvature suitable for the formation of polyanionic cage clusters. Second, hyperpolarizable oxyanions such as borate, tellurite, and plumbite, can interconnect to form numerous arrays of polyanionic structures, thereby making plumbite potential fundamental building block for synthesizing new POMs.^7^

Guided by the aforementioned strategy, we have succeeded in synthesizing a new family of POMs built from oxoanions of the carbon group element Pb with two distinct topologies, [Ln_6_(OH)_8_(H_30_Pb_18_O_32_)]·(ClO_4_)_12_·(H_2_O)_6_ (**LnPbOClO_4_‐1**, Ln = Y, Sm to Ho) and [Ln_6_(OH)_8_(H_32_Pb_18_O_32_)]_0.5_[Ln_6_(OH)_8_(H_2_O)_6_(H_24_Pb_12_O_24_)]_0.5_·(ClO_4_)_12_·(H_2_O)_6_ (**LnPbOClO_4_‐2**, Ln = Er and Tm), with the transition point existing between Ho and Er (**Figure** **1**a). Furthermore, the combination of rare‐earth or actinide ions with hyperpolarizable oxoanions, as well as dissociate charge‐balancing anions often yields cationic structures with potential anion‐exchange capacity, as exemplified by [ThB_5_O_6_(OH)_6_][BO(OH)_2_]·2.5H_2_O, [Th(MoO_4_)(H_2_O)_4_Cl]Cl·H_2_O, [Ce_2_Te_7_O_17_]X_2_ (X = Cl and Br), [M_2_Te_4_O_11_]X_2_ (M = Pu, Ce, and Zr; X = Cl or Br), etc.^8^ **YPbOClO_4_‐1** and **ErPbOClO_4_‐2** represent another example of such combination and they exhibit superior sorption capacity and selectivity toward ReO_4_^−^, which is a chemical surrogate of ^99^TcO_4_^−^, a predominate species for one of the most problematic radionuclides in the nuclear fuel cycle, ^99^Tc.^9^

**Figure 1:**
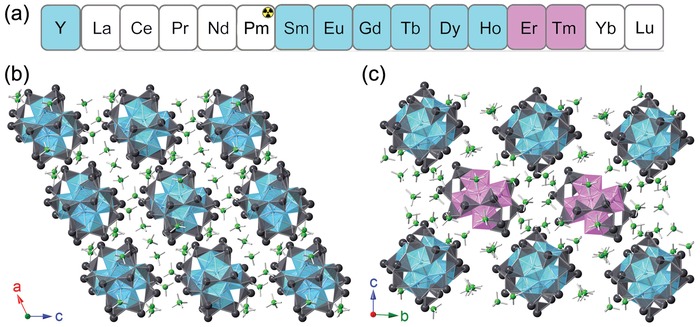
a) Periodic trend for the formation of lanthanide plumbite perchlorate. b) Depiction of the crystal structure of **LnPbOClO_4_‐1** (Ln = Y, Sm to Ho). c) Depiction of the crystal structure of **LnPbOClO_4_‐2** (Ln = Er and Tm). The Ln polyhedra are shown in blue or pink, Pb polyhedra in gray, and Cl atoms are in green.

Hydrothermal reactions between Ln_2_O_3_, PbO, and HClO_4_ at 220 °C resulted in the formation of two distinct phases, **LnPbOClO_4_‐1** and **LnPbOClO_4_‐2**, with the transition point existing between Ho and Er (Figure 1a). Such phase transition could be attributed to the lanthanide contraction, which increases the strain of the crystal lattice with the decreasing ionic radius. Similar effect has been observed in other systems including lanthanide borate, lanthanide tellurite sulfate, and lanthanide iodate selenate.^10^ An identical reaction was conducted on the lanthanide analog Y and it adopts the same structural type as that of Ho rather than Er, which is consistent with the periodic trend based on the ionic radius (Y 1.019 Å > Ho 1.015 Å > Er 1.004 Å, C.N. = 8).^11^

Single‐crystal X‐ray diffraction (XRD) analysis revealed that **LnPbOClO_4_‐1** and **LnPbOClO_4_‐2** crystallize in the monoclinic *C*2/*c* space group and triclinic space group $P\bar{1}$, respectively (Table S1, Supporting Information). The extended structures of both phases contain discrete cationic [Ln_6_O_8_]‐encapsulating polyplumbites clusters (denoted Ln_6_@Pb_18_ and Ln_6_@Pb_12_) with ClO_4_^−^ residing within the intermolecular spacing as counterions. **LnPbOClO_4_‐1** exclusively consists of Ln_6_@Pb_18_ units while **LnPbOClO_4_‐2** comprises both Ln_6_@Pb_18_ and Ln_6_@Pb_12_ moieties (Figure 1b,c). The structures of Ln_6_@Pb_18_ and Ln_6_@Pb_12_ can be best described as a [Pb_18_O_32_] nanosphere and a [Pb_12_O_24_] crown, respectively, where the [Ln_6_O_8_] ions are closely embedded in the cages of their corresponding topologies via the Pb−O−Ln bonds (**Figure** **2**a,b). Both assemblies are unprecedented in terms of constituent and structure, and they represent a brand new family (Group 14 element) of POMs. The 18‐core nanospheres are composed of nine crystallographically independent PbO_3_^4−^ trigonal‐pyramids through the $\bar{1}$ symmetry operation and they are approximately 11.7 Å in diameter as defined by the longest Pb···Pb distances. The 12‐core crowns consist of six crystallographically unique PbO_3_^4−^ polyhedra and they have a diameter of 11.7 Å as well. Remarkably, a close examination of the assembling of both moieties reveals that [Pb_12_O_24_] in fact is a secondary building unit of [Pb_18_O_32_]. The [Pb_18_O_32_] nanosphere can be considered as being constructed from a [Pb_12_O_24_] crown capped by two Pb_3_ rings from its polar sites (Figure 2c). Such diversity in self‐assembling implies the richness of structural chemistry of polyplumbite and the potential of creating new Pb POMs with different number of vertexes. All the PbO_3_^4−^ polyhedra are corner‐shared via the Pb−O−Pb bridges with the Pb−O distances ranging from 2.2 to 2.4 Å and the lone‐pair electrons of PbO_3_^4−^ point toward the intermole‐cular space, which creates corrugated geometries suitable for the formation of cage clusters. Similarly, the uranyl−peroxide−uranyl bridges have dihedral angles of ≈140° and this curvature promotes the formation of uranyl peroxide cage clusters.[qv: 3a]

**Figure 2:**
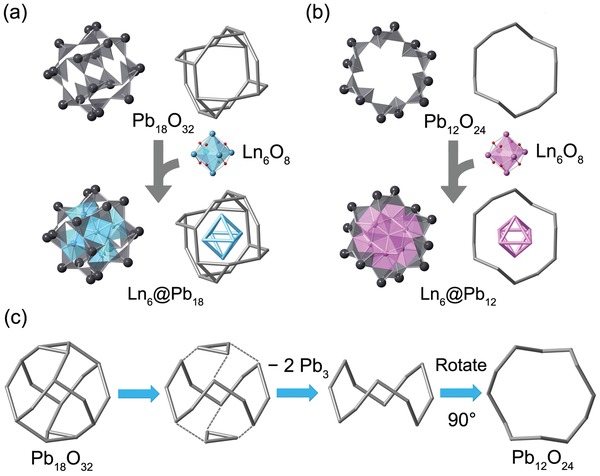
a) Combined polyhedral and ball‐and‐stick representations of the Ln_6_@Pb_18_ cluster in **LnPbOClO_4_‐1** and **LnPbOClO_4_‐2**. b) Combined polyhedral and ball‐and‐stick representations of the Ln_6_@Pb_12_ clusters in **LnPbOClO_4_‐2**. c) Stick representations showing the structural correlation between [Pb_18_O_32_] nanosphere and [Pb_12_O_24_] crown. The Ln polyhedra are shown in blue or pink, and Pb polyhedra in gray.

While heteropolymetalates containing mononuclear heteroatom in the center of anions are well documented as in the Keggin and Dawson structures, polynuclear cores encapsulating POMs are relatively rare.^12^ Examples of these include [Ta_12_]/[Ta_16_] cluster‐containing polytantalotungstates, [Bi_6_O_8_]/[Pb_8_O_6_]‐containing U_24_ POMs, [K_42_(H_2_O)_60_]‐containing polytungstate, etc.[qv: 4a,c,13] **LnPbOClO_4_‐1** and **LnPbOClO_4_‐2** represent another unusual case of POMs with [Ln_6_O_8_] hexanuclear cores embedded. The Ln hexamers in Ln_6_@Pb_18_ and Ln_6_@Pb_12_ have an identical topology and six Ln metal centers bridged by eight *µ*_3_‐OH ligands forming an octahedral geometry. Similar motifs have been observed for other trivalent lanthanides and a wide variety of tetravalent metals.^14^ It is noteworthy to mention that another hexanuclear form of lanthanides is Ln_6_(*µ*_6_‐O)(*µ*_3_‐OH)_8_, which has a *µ*_6_‐oxo bridge at the center of the assembly, but the Ln_6_(*µ*_3_‐OH)_8_ in this study suggests otherwise.^15^ All the Ln centers are eight‐coordinate with a square antiprism geometry and the Ln−O distances vary from 2.3 to 2.5 Å.

Considering the cationic nature of the Ln‐Pb clusters and the low affinity of ClO_4_^−^ anions to the cationic moieties, **LnPbOClO_4_‐1** and **LnPbOClO_4_‐2** are well suited for anion‐exchange studies especially with ReO_4_^−^, which has a similar coordination geometry as that of ClO_4_^−^. **YPbOClO_4_‐1** and **ErPbOClO_4_‐2** were chosen as the model absorbents since they have the smallest molecular weights (corresponding to highest adsorption capacity in mg g^−1^) in their respective structure types, as well as high yields and purities (Figure S1, Supporting Information). Scanning electron microscopy (SEM) and energy‐dispersive spectroscopy (EDS) analyses suggest that anion exchange between ReO_4_^−^ and ClO_4_^−^ can take place in both materials (**Figure** **3**a,b and Figure S2, Supporting Information). The exchange process was further confirmed by the Fourier‐transform infrared spectroscopy spectra of ReO_4_^−^ soaked crystals, showing the emerging Re−O ν_3_ asymmetric stretching bands (≈890 cm^−1^) and concomitantly reduced intensity of Cl−O ν_1_ stretching bands (≈1050 cm^−1^) (Figure S3, Supporting Information). The ReO_4_^−^ ion‐exchange kinetics of **YPbOClO_4_‐1** and **ErPbOClO_4_‐2** were conducted with initial Re concentration of 400 mg L^−1^ at a solid/liquid ratio of 1 g L^−1^ at 300 K. As shown in Figure 3c, **YPbOClO_4_‐1** and **ErPbOClO_4_‐2** are able to remove up to 76.0% and 95.2% of ReO_4_^−^ from the solution within 24 h, respectively, both of which are significantly higher than the removal rates of other cationic inorganic materials, e.g., Yb_3_O(OH)_6_Cl (7.7%) and Mg‐Al LDH (40.3%), under the same sorption conditions.^16^ The effect of pH on ReO_4_^−^ sorption was investigated, showing that **YPbOClO_4_‐1** and **ErPbOClO_4_‐2** exhibit highest sorption of ReO_4_^−^ under pH 6 (267 mg g^−1^) and pH 8 (294 mg g^−1^), respectively (Figure 3d). To measure the maximum sorption capacities, sorption as a function of molar ratio of ReO_4_^−^/**LnPbOClO_4_** was conducted as shown in Figure 3e. The sorption was saturated when molar ratio of ReO_4_^−^/ClO_4_^−^ increased up to 5:1 or 10:1 and the maximum sorption capacities of ReO_4_^−^ by **YPbOClO_4_‐1** and **ErPbOClO_4_‐2** were 434.7 and 427.7 mg g^−1^, respectively, which are close to the theoretical value of 480.4 and 493.9 mg g^−1^ for the corresponding materials, assuming the ClO_4_^−^ anions are fully substituted by ReO_4_^−^. Comparing with other reported inorganic sorbents for ReO_4_^−^ removal, the sorption capacities of **YPbOClO_4_‐1** and **ErPbOClO_4_‐2** are approximately one order of magnitude larger than that of biochar (46.5 mg g^−1^) and Yb_3_O(OH)_3_Cl (48.6 mg g^−1^), and nearly three times the highest reported value 162 mg g^−1^ for NDTB‐1 (Table S2, Supporting Information).[qv: 16a,17] In fact, the sorption capacities of **YPbOClO_4_‐1** and **ErPbOClO_4_‐2** are notably higher than most of cationic metal‐organic frameworks (MOFs) including the recently reported Tc‐uptake MOFs SCU‐101 and SCU‐102.^18^ This can be partially attributed to the high positive charge density of the Ln_6_@Pb_18_ and Ln_6_@Pb_12_ clusters. Since the concentration of NO_3_^−^ in the high‐level nuclear waste solutions is much higher than that of TcO_4_^−^ (e.g., molar ratio equals to 314 at Hanford site), the competing effect of NO_3_^−^ on the sorption of TcO_4_^−^ is critical for the purpose of practical application. The competing ion exchange experiments of ReO_4_^−^ (0.15 × 10^−3^ m) by **YPbOClO_4_‐1** and **ErPbOClO_4_‐2** were conducted in the presence of various amounts of NO_3_^−^. As shown in Figure 3f, **YPbOClO_4_‐1** and **ErPbOClO_4_‐2** can still capture approximately 84% and 83% of ReO_4_^−^, respectively, from aqueous solution with up to 100 times excess of NO_3_^−^ as a competing anion. **YPbOClO_4_‐1** and **ErPbOClO_4_‐2** exhibit excellent exchange selectivity toward ReO_4_^−^ over other anions, including SO_4_^2−^, PO_4_^3−^, CO_3_^2−^, Cl^−^, and B(OH)_4_^−^ (Section S1.2.4 and Figure S4, Supporting Information). These results indicate that **YPbOClO_4_‐1** and **ErPbOClO_4_‐2** have successfully overcome the critical drawbacks of low sorption capacity and poor selectivity for traditional inorganic anion‐exchange materials toward ReO_4_^−^/TcO_4_^−^ sequestration.

**Figure 3:**
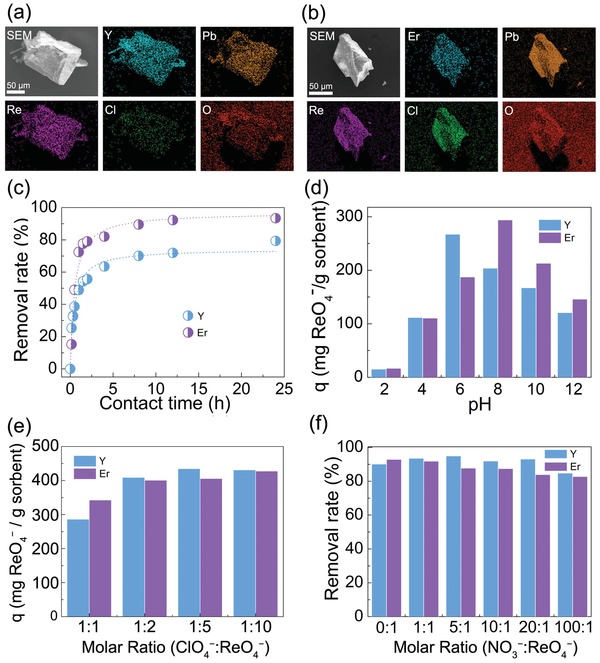
a) SEM–EDS mapping of ReO_4_^−^‐soaked **YPbOClO_4_**. b) SEM–EDS mapping of ReO_4_^−^‐soaked **ErPbOClO_4_**. c) Sorption kinetics of ReO_4_^−^ by **YPbOClO_4_** and **ErPbOClO_4_** at pH 8. d) Effect of pH (2−12) on the removal rate of ReO_4_^−^. e) Sorption capacities of **YPbOClO_4_** and **ErPbOClO_4_**. f) Effect of NO_3_^−^ on the adsorption of ReO_4_^−^.

The original shape of **YPbOClO_4_‐1** and **ErPbOClO_4_‐2** crystals retained well after sorption of ReO_4_^−^, however, they transformed from transparent single crystals to opaque crystals, suggesting that both materials underwent a solvent‐mediated anion exchange with a possible recrystallization process (**Figure** **4**a). Powder XRD studies indicate a phase transition occurs after the ReO_4_^−^ adsorption (Figures S1 and S5, Supporting Information). By breaking ion‐exchanged products into small crystallites, single crystals of ReO_4_^−^ adsorbed product [Er_6_(*µ*_3_‐OH)_8_(H_30.25_Pb_18_O_32_)]·(ReO_4_)_12.25_·(H_2_O)*_x_* (**ErPbOReO_4_**) were obtained and a single crystal‐to‐single‐crystal structural transformation mechanism was elucidated for the sorption of ReO_4_^−^. All examined **ErPbOReO_4_** crystals had poor quality and the final structural refinements were less than satisfactory, but the Ln–Pb moieties and ReO_4_^−^ anions can be accurately located on the electron density map. **ErPbOReO_4_** crystalizes in the same space group $P\bar{1}$ as **ErPbOClO_4_‐2**, but with different unit cell parameters and structure (Table S3, Supporting Information). It is composed of solely cationic Ln_6_@Pb_18_ nanospheres with ReO_4_^−^ residing within the intermolecular spacing as shown in Figure 4b. Surprisingly, the Ln_6_@Pb_12_ crowns in **LnPbOClO_4_‐2** transferred to Ln_6_@Pb_18_ nanospheres in **ErPbOReO_4_**. Correspondingly, six crystallographically independent Er and 18 unique Pb atoms are located in **ErPbOReO_4_**, rather than six Er and 15 Pb atoms found in **LnPbOClO_4_‐2**. The presence of ReO_4_^−^ in **ErPbOReO_4_** can be simply differentiated from ClO_4_^−^ by the longer Re−O bond distances (1.6–1.7 Å) than those of Cl−O (1.4–1.5 Å). A close inspection of the coordination environment of ReO_4_^−^ suggests a secondary covalent interaction between lead centers and ReO_4_^−^ anions (Figure 4c), with Pb^2+^···O‐ReO_3_^−^ distances ranging from 2.70(2) to 2.75(2) Å (Table S4, Supporting Information), which slightly exceed the accepted distance of Pb−O covalent bond 2.60(19) Å but are well within the range of secondary Pb–O bond distances.^19^ The longer Re−O bond length (1.6–1.7 Å) versus that of Cl−O (1.4–1.5 Å) in ClO_4_^−^ and N−O (1.2 to 1.3 Å) in NO_3_^−^ enable shorter Pb^2+^···oxoanion distance and notably stronger interaction between Pb^2+^ and ReO_4_^−^ (Tables S5, S6, and S7, Supporting Information). This interaction offers the inherent driving force for the spontaneous diffusion of ReO_4_^−^ anions toward the cationic moieties and concomitantly structural transformation, as well as for the remarkable uptake selectivity toward ReO_4_^−^. A similar anion exchange mechanism between the carboxylates and the disulfonate ions in a 2D cationic layered material [Pb_2_F_2_][O_3_SCH_2_CH_2_SO_3_] (SLUG‐32) has been observed due to a stronger lead–carboxylate interaction.[qv: 19a]

**Figure 4:**
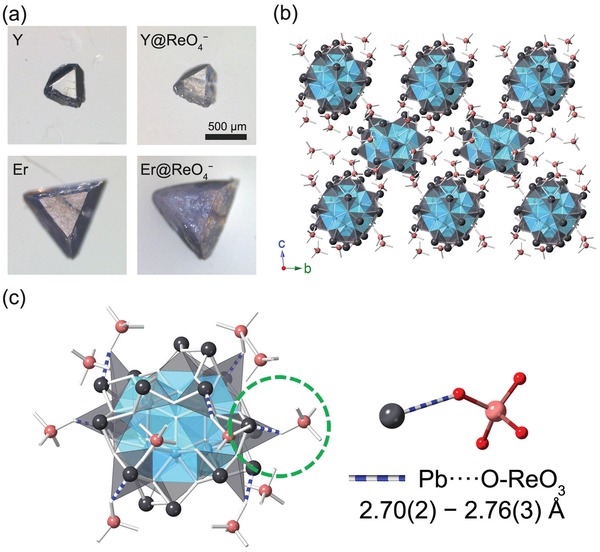
a) Optical micrographs of **YPbOClO_4_‐1** and **ErPbOClO_4_‐2** before and after ReO_4_^−^ sorption. b) Depiction of the crystal structure of **ErPbOReO_4_**. c) Depiction of the Pb^2+^···O‐ReO_3_^−^ bonding in **ErPbOReO_4_**. The Er polyhedra are shown in blue, Pb atoms in gray, and Re atoms are in light red.

In conclusion, we present here a new family of POMs composed of [Pb_18_O_32_] and [Pb_12_O_24_] polyanionic structures with [Ln_6_O_8_] clusters encapsulated within the moieties. The corrugated geometry of plumbite created by the stereochemically active lone‐pair electrons on the Pb center and the high tendency of forming polyanionic structures are beneficial to further development of POM materials. **YPbOClO_4_‐1** and **ErPbOClO_4_‐2** have the potential of selectively trap TcO_4_^−^ in nuclear waste owing to their exceptional adsorption capacities toward ReO_4_^−^ (434.7 and 427.7 mg g^−1^), which are the highest for all inorganic materials. The adsorption mechanism is directly visualized by the single‐crystal structure of ReO_4_^−^ adsorbed material **ErPbOReO_4_**. Further investigation of this and related types of POMs is underway to develop a practical solution for sequestrations of anionic radioactive pollutants, e.g., ^99^TcO_4_^−^.

## Experimental Section

*Materials*: Ln_2_O_3_ (Ln = Y, Sm, Eu, Gd, Dy, Ho, Er, and Tm) (99.9%, Aladdin), Tb_4_O_7_ (99.9%, Aladdin), PbO (99%, Aladdin), and HClO_4_ (70%, Aladdin) were used as received. Distilled and Millipore‐filtered water with a resistance of 18.2 MΩ cm was used in all reactions.

*Synthesis*: 0.5 mmol Ln_2_O_3_ or 0.25 mmol Tb_4_O_7_ (0.1744 g for Sm_2_O_3_, 0.1760 g for Eu_2_O_3_, 0.1813 g for Gd_2_O_3_, 0.1869 g for Tb_4_O_7_, 0.1865 g for Dy_2_O_3_, 0.1889 g for Ho_2_O_3_, 0.1913 g for Er_2_O_3_, and 0.1929 g for Tm_2_O_3_), PbO (1.5 mmol, 0.1196 g), and 1 m HClO_4_ solution (2 mmol, 2 mL) were loaded into a PTEF‐lined Parr 4749 autoclave with a 25 mL internal volume. The autoclaves were sealed and heated to 220 °C for 4 days and were cooled to room temperature at a rate of 5 °C h^−1^. The reaction products were washed with deionized water to remove soluble solids, followed by rinsing with ethanol. Tablets of [Ln_6_(OH)_8_(H_30_Pb_18_O_32_)]·(ClO_4_)_12_·(H_2_O)_6_ (**LnPbOClO_4_‐1**, Ln = Sm to Ho, Y) and [Ln_6_(µ_3_‐OH)_8_(H_32_Pb_18_O_32_)]_0.5_[Ln_6_(*µ*_3_‐OH)_8_(H_2_O)_6_(H_24_Pb_12_O_24_)]_0.5_·(ClO_4_)_12_·(H_2_O)_6_ (**LnPbOClO_4_‐2**, Ln = Er and Tm) were isolated as pure phases with yield ranging from 30% to 62%, respectively, based on Ln (Figure S6, Supporting Information). SEM–EDS demonstrated the presence of Ln, Pb, Cl, and O in the crystals (Figure S7, Supporting Information).

*X‐Ray Crystallography Studies*: Single‐crystal data of **LnPbOClO_4_‐1**, **LnPbOClO_4_‐2**, and **ErPbOReO_4_** were collected on a Bruker D8‐Venture single‐crystal X‐ray diffractometer equipped with a Turbo X‐ray Source (Mo Kα radiation, λ = 0.71073 Å) adopting the direct‐drive rotating‐anode technique and a complementary metal oxide semiconductor detector at 173 K. The data frames were collected using the program *APEX2* and processed using the *SAINT* routine in *APEX2*.^20^ The structures were solved by direct methods and refined by the full‐matrix least squares on F^2^ using the *SHELXTL‐2014* program.^21^ All non‐H atoms were refined with anisotropic displacement parameters. Considering the electroneutrality of the structure and the bond valence sum (BVS) calculations using measured bond distances, the hexanuclear [Ln_6_O_8_] cores in **LnPbOClO_4_‐1**, **LnPbOClO_4_‐2**, and **ErPbOReO_4_** were defined as the [Ln_6_(*µ*_3_‐OH)_8_] with protonated O atoms distributed on the nodes of the octahedra. Similar hexanuclear cores were reported for Tb/Eu‐based metal‐organic frameworks (DMA)_2_[Eu_6_ *_x_*Tb_6(1−_ *_x_*_)_(*µ*_3_‐OH)_8_(BPDC)_6_]·*x*(solvent).[qv: 14f] Since **LnPbOClO_4_‐1** and **LnPbOClO_4_‐2** were synthesized under acid conditions, the O atoms of plumbite anions were considered to be partially or fully protonated to ensure the electroneutrality of overall structures, which could be further supported by the BVS calculations (Tables S7 and S8, Supporting Information). Selected crystallographic information is listed in Tables S1 and S3 in the Supporting Information. Atomic coordinates and additional structural information are provided in the crystallographic information files.

## Conflict of Interest

The authors declare no conflict of interest.

## Supporting information

Supplementary
