# Supplementary material for: [Ln6O8] Cluster‐Encapsulating Polyplumbites as New Polyoxometalate Members and Record Inorganic Anion‐Exchange Materials for ReO4 − Sequestration
Source: Adv Sci (Weinh). 2019 Jun 17;6(17):1900381. doi: 10.1002/advs.201900381 (PMC6724469; doi:10.1002/advs.201900381)
Supplement: Supplementary file 1 — Supplementary [file ADVS-6-1900381-s001.pdf]

Copyright WILEY-VCH Verlag GmbH & Co. KGaA, 69469 Weinheim, Germany, 2019.

ADVANCED SCIENCE
Open Access

Supporting Information

for *Adv. Sci.,* DOI: 10.1002/advs.201900381

[Ln_6O_8] Cluster-Encapsulating Polyplumbites as New Polyoxometalate Members and Record Inorganic Anion-Exchange Materials for ReO_4^- Sequestration

Jian Lin, Lin Zhu, Zenghui Yue, Chuang Yang, Wei Liu, Thomas E. Albrecht-Schmitt, Jian-Qiang Wang, and Shuao Wang**

Copyright WILEY-VCH Verlag GmbH & Co. KGaA, 69469 Weinheim, Germany, 2019.

Supporting Information

[Ln_6O_8] Cluster-Encapsulating Polyplumbites as New Polyoxometalate Members and Record Inorganic Anion-Exchange Materials for ReO_4^- Sequestration

Jian Lin, Lin Zhu, Zenghui Yue, Chuang Yang, Wei Liu, Thomas E. Albrecht-Schmitt, Jian-Qiang Wang and Shuao Wang**

S1. EXPERIMENTAL SECTION

S1.1 Characterizations and Methods

S1.1.1 Powder X-ray diffraction (PXRD) Studies. PXRD patterns were collected from 5° to 50° with a step of 0.02° using a Bruker D8 Advance X-ray diffractometer with Cu Kα radiation (λ = 1.54056 Å) equipped with a Lynxeye one-dimensional detector.

S1.1.2 Elemental Analysis

Scanning Electron Microscopy (SEM) and Energy-Dispersive Spectroscopy (EDS) Analysis. SEM images and EDS data were recorded on a Zeiss Merlin Compact LEO 1530 VP scanning electron microscope. The energy of the electron beam voltage was 10 keV for imaging and was 15 keV for quantitative identifications of elements. Samples were attached directly on the carbon conductive tape. The spectra acquisition time was 60 s and all EDS results are provided in Figure S2 and Figure S7.

ICP-OES Analysis. To determine the molar ratio of Ln/Pb in **$YPbOClO_4$-1** and **$ErPbOClO_4$-2**, 5 mg of samples were dissolved in concentrated HNO_3 by multiple dissolution and wash process and the final volume of solutions was adjusted to 100 mL. The concentration of Y/Er and Pb in aqueous solutions were determined by ICP-OES, showing that **$YPbOClO_4$-1** has a Y/Pb molar ratio of 3.27 (*Calcd.* 3 %) and **$ErPbOClO_4$-2** has an Er/Pb molar ratio of 2.69 (*Calcd.* 2.5).

S1.1.3 Fourier Transform Infrared (FTIR) Spectrum. The FTIR spectra were recorded in the range of 400−4000 cm^{-1} on a Thermo Nicolet 6700 spectrometer equipped with a diamond attenuated total reflectance (ATR) accessory. The presence of water and OH groups in **$YPbOClO_4$-1** and **$ErPbOClO_4$-2** can be confirmed by the O−H stretching bands ranging from 3,000−3,600 cm^{-1} (Figure S3). The bending mode of the H−O−H can be located approximately at ~1,620 cm^{-1} for both complexes. The vibrational spectra of ClO_4^- show bands appearing at 1050 cm^{-1} (v_1 stretching) and 610 cm^{-1} (v_4 bending mode). **$YPbOClO_4$-1** and **$ErPbOClO_4$-2** were recovered after sorption studies by countrification and washing with ethanol then drying in ambient condition. The ReO_4^--soaked **$YPbOClO_4$-1** and **$ErPbOClO_4$-2** show Re–O v_3 stretching vibration bands at ~890 cm^{-1}.

S1.1.4 Thermogravimetric analysis (TGA). TGA of **$YPbOClO_4$-1** and **$ErPbOClO_4$-2** was carried out on a NETZSCH STA 449F3 instrument in the range of 25 − 900 °C at a heating rate of 10 °C min^{-1} under a nitrogen flow. As shown in Figure S8, **$YPbOClO_4$-1** and **$ErPbOClO_4$-2** experience similar processes in weight loss due to their same molecular component and identical structure. **$YPbOClO_4$-1** and **$ErPbOClO_4$-2** have an initial weight loss of ~7% and ~9% upon heating from 25 to 300°C, respectively, which can be attributed to the loss of hydrating water molecules or $HClO_4$. The sharp mass loss weight of ~12% for **$YPbOClO_4$-1** and ~13% for **$ErPbOClO_4$-2** occurring between 300 to 400 °C could be originating from the further loss of $HClO_4$. The Ln_6Pb_{18} or Ln_6Pb_{12} cores in **$YPbOClO_4$-1** and **$ErPbOClO_4$-2** appear to be stable up to 600 °C and 800 °C, respectively.

1.1.5. X-ray Photoelectron Spectroscopy (XPS) Studies. The XPS data of **$YPbOClO_4$-1** and **$ErPbOClO_4$-2** were recorded on a Thermo SCIENTIFIC ESCALAB 250Xi spectrometer using monochromatic Al Kα

(1486.8 eV) X-ray radiation at room temperature. The anode was operated at 15 kV and 10mA with a typical spot size of 500 µm. As shown in Figure S9, the binding energies (BEs) of Cl $2p_{1/2}$, Cl $2p_{3/2}$, Pb $4f_{5/2}$, and Pb $4f_{7/2}$ for both compounds, as referenced to adventitious C 1s (BE = 285.0 eV), are 209.5, 208.0, 143.7, 138.8 eV, respectively, which agree well with the values for perchlorate[1] and plumbite[2]. In addition, the trivalent nature of Y and Er in **$YPbOClO_4$-1** and **$ErPbOClO_4$-2**can be confirmed by Y $3d_{3/2}$/Y $3d_{5/2}$ and Er 4d bands at 161.0/158.0 and 168.5 eV respectively.[3]

S1.2 Anion Exchange Studies. All the experiments were conducted under ambient conditions using the batch sorption method. The solid/liquid ratio performed in all batch experiments was 1 g L^{-1}. In a typical experiment, 10 mg of **$YPbOClO_4$-1** or **$ErPbOClO_4$-2** was added into 10 mL of aqueous solution containing certain contents of ReO_4^-. The resulting mixture was stirred for a desired contact time and separated with a 0.22 µm nylon membrane filter. The concentrations of ReO_4^- in aqueous solution were determined by inductively coupled plasma-mass spectrometry (ICP-MS, Thermo Scientific) and inductively coupled plasma optical emission spectrometry (ICP-OES, Thermo Fisher Scientific iCAP 7000).

S1.2.1 Exchange kinetics studies. 100 mg of **$YPbOClO_4$-1** or **$ErPbOClO_4$-2** material was added into 100 mL of a solution containing 28 mg L^{-1} ReO_4^-. The resulting mixture was stirred for a desired contact time. The concentrations of ReO_4^- in aqueous solution were determined by ICP-OES. The removal ratios (R) of perrhenate were calculated using $R = (C_0 - C_t)/C_0 \times 100\%$ (where C_0 and C_t represent the initial concentration and concentration at time t, respectively). Sorption kinetics of ReO_4^- were fitted to the pseudo-first-order kinetic model and the pseudo-second-order kinetics model, $\ln(q_e - q_t) = \ln q_e - k_1 t$ and $t/q_t = 1/h + t/q_e$, respectively (where q_t, q_e represent the amounts of adsorbate at certain time t or at equilibrium time, h is the initial adsorption rate, $h = k_2 q_e^2$, k_1 and k_2 are the sorption rate constants) (Table S7).

S1.2.2 Effect of pH studies. The sorbents after immersed into aqueous solutions containing 400 mg L^{-1} ReO_4^- with different pH values ranging from 2 to 12 and stirred for 12 hours, the suspension was

separated with a 0.22 μm nylon membrane filter and the concentrations of ReO_4^- after sorption in aqueous solution were determined by ICP-OES.

S1.2.3 Adsorption capacity studies. The molar ratio sorption experiments of ReO_4^- by **YPbOClO₄-1** or **ErPbOClO₄-2** were performed under different molar ratios of ClO_4^- (**YPbOClO₄-1** or **ErPbOClO₄-2**) / ReO_4^- varying from 1:1 to 1:20. 10 mg of **YPbOClO₄-1** or **ErPbOClO₄-2** was added into 10 mL of aqueous solution containing certain molar ratio of ClO_4^- (**LnPbOClO₄**) / ReO_4^- and stirred for 12 hours, the concentrations of ReO_4^- after sorption in aqueous solution were determined by ICP-OES.

S1.2.4 Anion exchange selectivity studies. The effect of NO_3^- was performed by adding 0.15 mM, 0.75 mM, 1.5 mM, 3 mM, or 15 mM $NaNO_3$ solutions into a 0.15 mM ReO_4^- solution. The competing effects of other anions including SO_4^{2-}, PO_4^{3-}, CO_3^{2-}, Cl^- and $B(OH)_4^-$ were performed by adding 0.5 mM Na_2SO_4, Na_2HPO_4, Na_2CO_3, NaCl or H_3BO_3 solutions into a 0.5 mM ReO_4^- solution. **YPbOClO₄-1** or **ErPbOClO₄-2** were added in the above solution and then the concentrations of ReO_4^- in aqueous solution after sorption were determined by ICP-OES (Figure S4 and Table S10).

S2. FIGURES AND TABLES

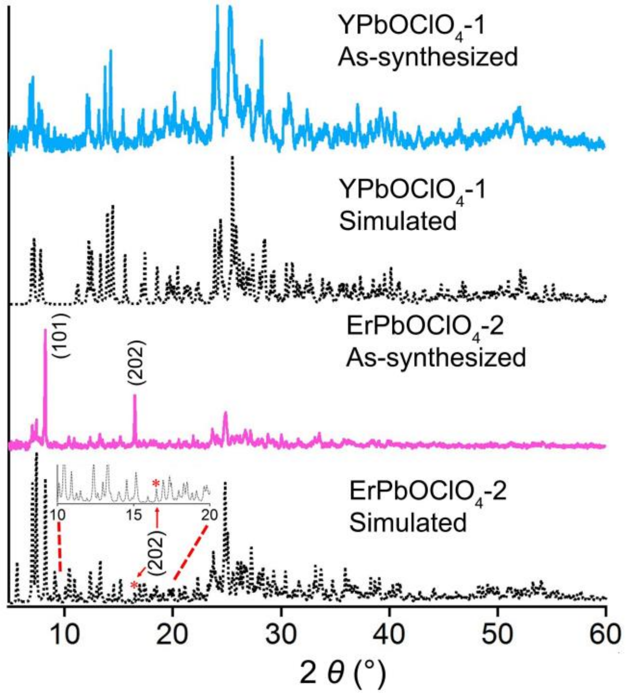

Figure S1. PXRD patterns of **YPbOClO$_4$-1** and **ErPbOClO$_4$-2** showing the phase purities of both materials.

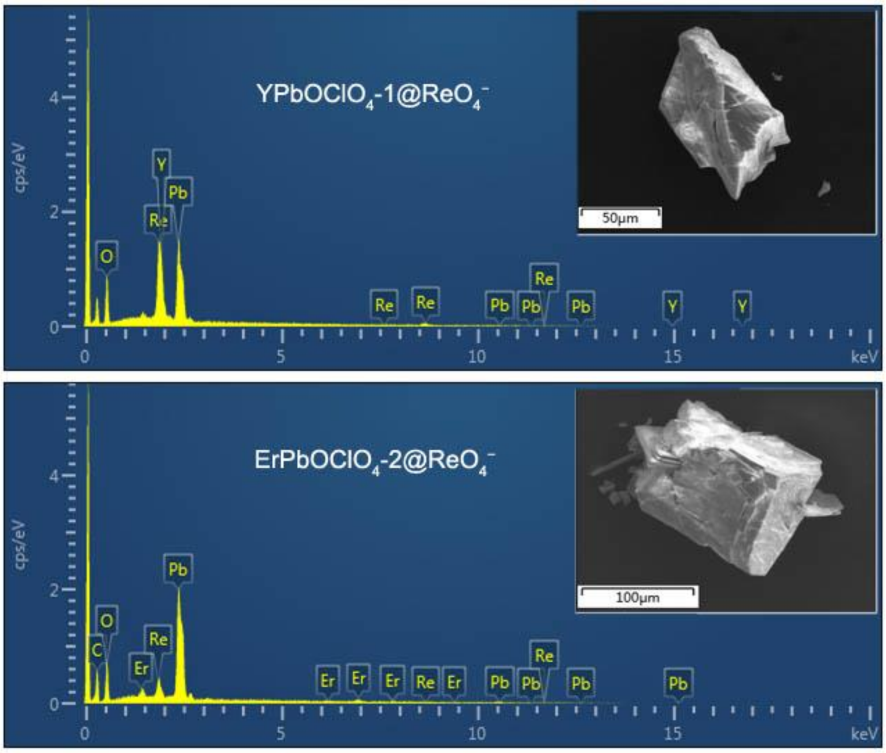

Figure S2. SEM images and EDS spectra of ReO_4^--adsorpted **YPbOClO$_4$-1** and **ErPbOClO$_4$-2**.

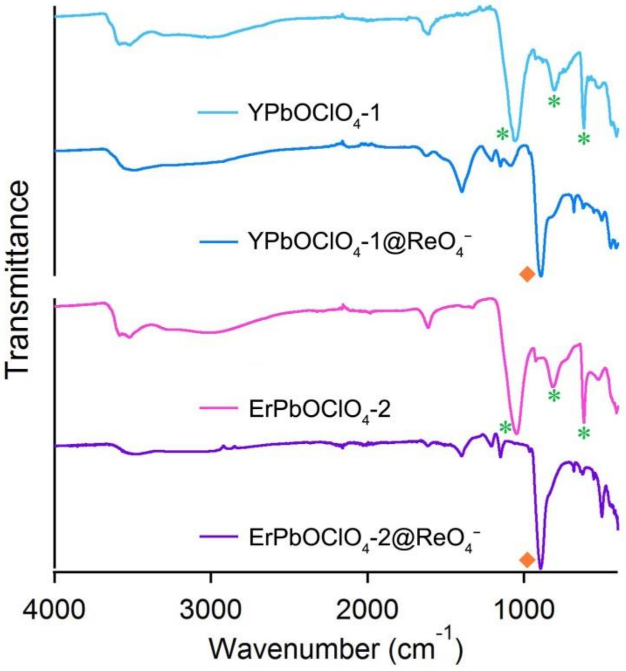

Figure S3. FTIR spectra of **YPbOClO$_4$-1** and **ErPbOClO$_4$-2** before and after ReO_4^- sorption. The vibrational bands of ClO_4^- and ReO_4^- are labled with (*) and with (◆, respectively.

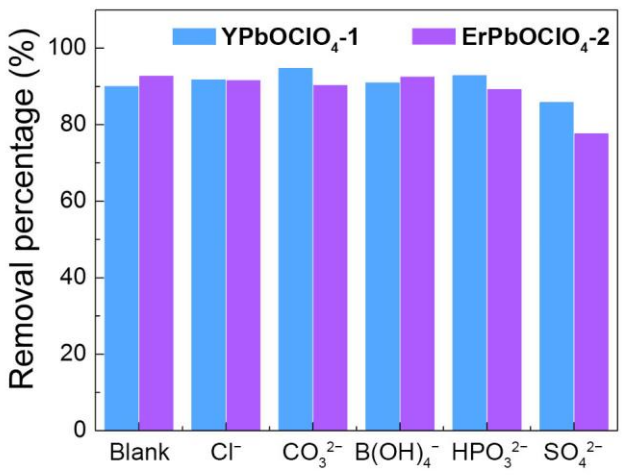

Figure S4. Effects of common anions on the removal percentage of ReO_4^- by **$YPbOClO_4$-1** and **$ErPbOClO_4$-2**.

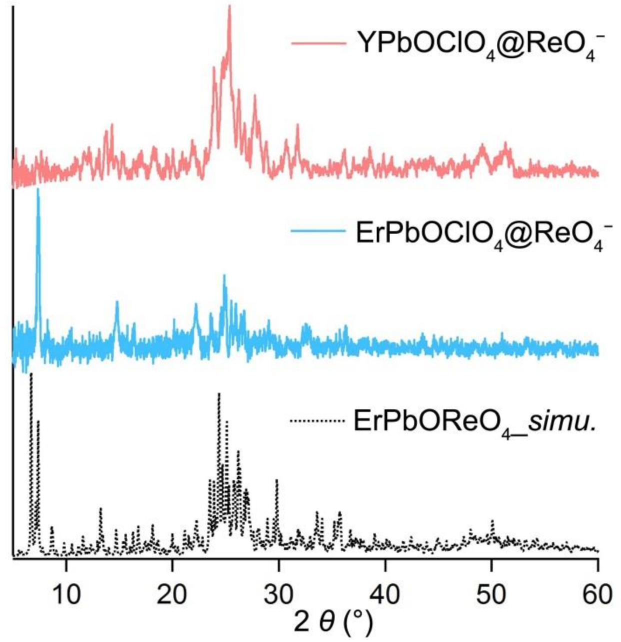

Figure S5. PXRD patterns of **$ErPbOClO_4$-1** and **$ErPbOClO_4$-2** after ReO_4^- sorption.

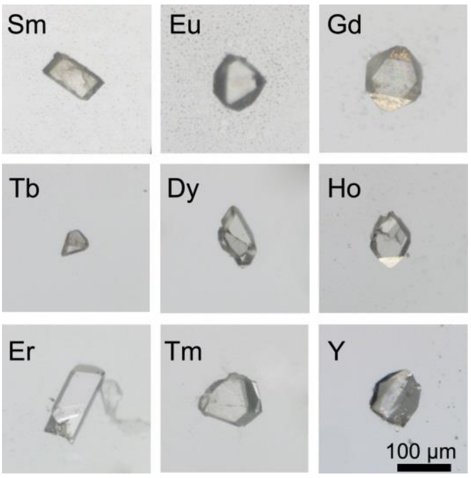

Figure S6. Crystal images of **$LnPbOClO_4$-1** (Ln = Sm to Ho) and **$LnPbOClO_4$-2** (Ln = Er and Tm).

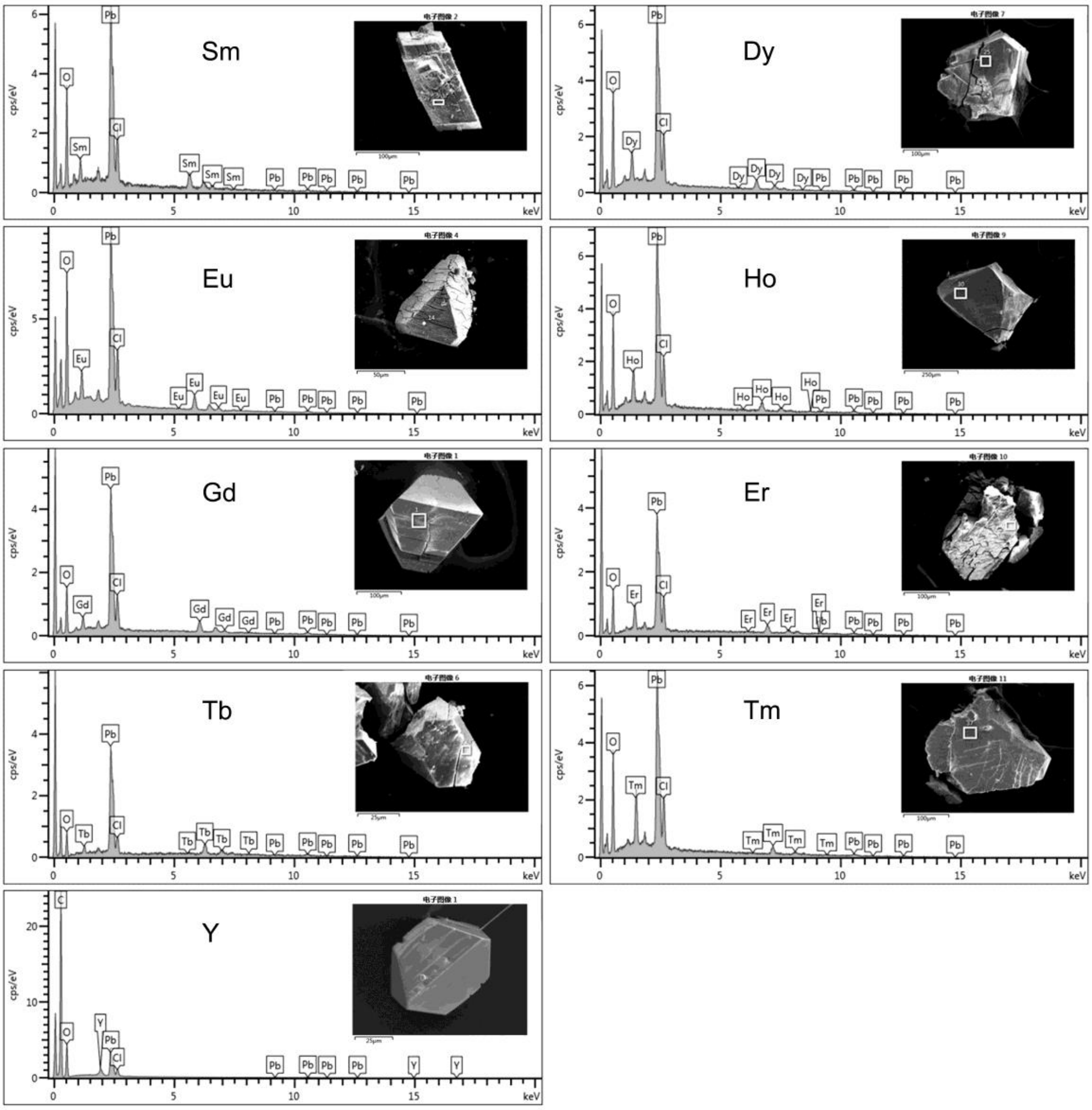

Figure S7. SEM images and EDS spectra of **LnPbOClO$_4$-1** (Ln = Sm to Ho, Y) and **LnPbOClO$_4$-2** (Ln = Er and Tm).

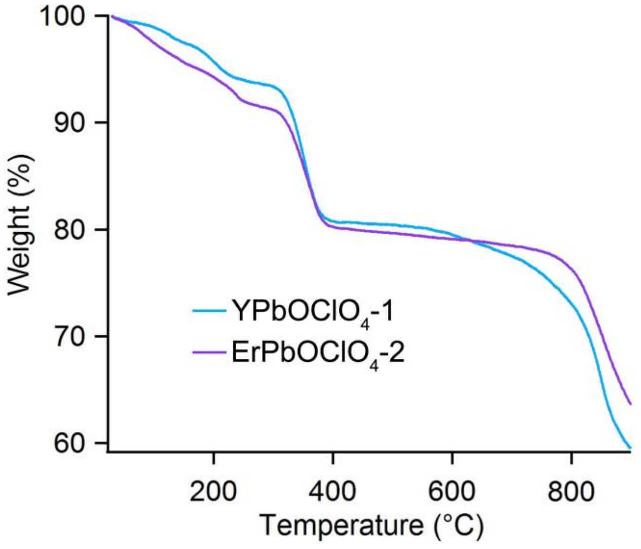

Figure S8. Thermogravimetric curves of **$YPbOClO_4$-1** and **$ErPbOClO_4$-2**.

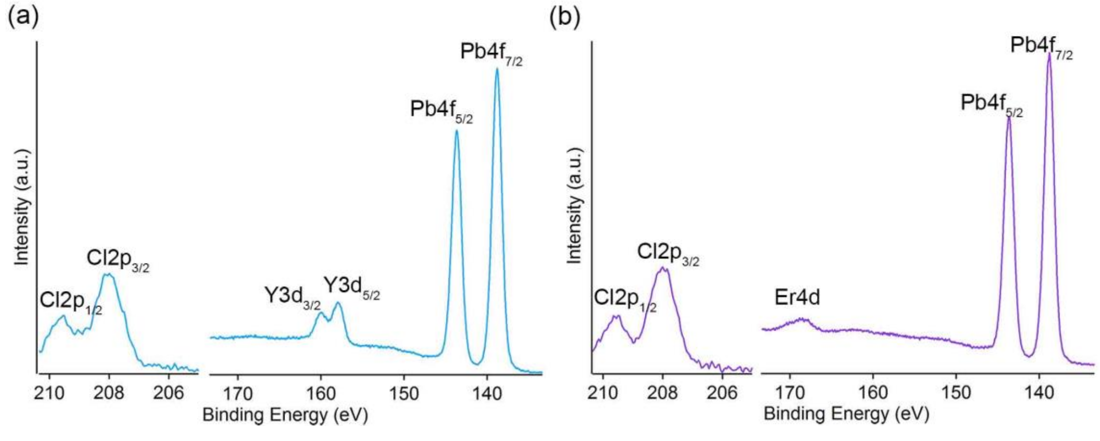

Figure S9. XPS spectra of (a) **$YPbOClO_4$-1** and (b) **$ErPbOClO_4$-2**.

Table S1. Crystallographic data for **$LnPbOClO_4$-1** (Ln = Sm to Ho, Y) and **$LnPbOClO_4$-2** (Ln = Er, Tm).

Compound	**Y**	**Sm**	**Eu**	**Gd**	**Tb**	**Dy**	**Ho**	**Er**	**Tm**
Formula Mass	$Y_6Pb_{18}Cl_{12}O_{94}$ 6192.28	$Sm_6Pb_{18}Cl_{12}O_{94}$ 6560.92	$Eu_6Pb_{18}Cl_{12}O_{94}$ 6570.58	$Gd_6Pb_{18}Cl_{12}O_{94}$ 6602.32	$Tb_6Pb_{18}Cl_{12}O_{94}$ 6612.34	$Dy_6Pb_{18}Cl_{12}O_{94}$ 6633.82	$Ho_6Pb_{18}Cl_{12}O_{94}$ 6648.40	$Er_6Pb_{15}Cl_{12}O_{93}$ 6024.81	$Tm_6Pb_{15}Cl_{12}O_{93}$ 6034.83
Color	Colorless	Colorless	Colorless	Colorless	Colorless	Colorless	Pink	Purple	Colorless
Habit	Tablet	Tablet	Tablet	Tablet	Tablet	Tablet	Tablet	Tablet	Tablet
Space Group	*C*2/*c*	*C*2/*c*	*C*2/*c*	*C*2/*c*	*C*2/*c*	*C*2/*c*	*C*2/c	$P\bar{1}$	$P\bar{1}$
a (Å)	25.158(2)	25.307(4)	25.319(3)	25.345(3)	25.3160(14)	25.271(2)	25.2969(15)	14.6180(15)	14.6169(9)
b (Å)	14.6633(12)	14.759(3)	14.736(2)	14.8081(16)	14.7914(8)	14.6956(13)	14.7727(8)	15.7837(18)	15.7516(11)
c (Å)	28.031(2)	28.260(6)	28.264(5)	14.8081(16)	28.3373(16	28.238(3)	28.2775(15)	19.706(2)	19.6429(11)
α (°)	90	90	90	90	90	90	90	91.871(4)	91.671(3)
β (°)	115.472(3)	115.117(5)	115.105(4)	114.988(3)	115.0178(17)	115.088(3)	115.1823(16)	99.316(4)	99.260(2)
γ (°)	90	90	90	90	90	90	90	94.749(4)	94.767(3)
V ($Å^3$)	9335.3(13)	9558(3)	9550(3)	9654(2)	9615.6(9)	9497.2(15)	9563.1(9)	4466.5(8)	4444.0(5)
Z	4	4	4	4	4	4	4	2	2
T (K)	173(2)	173(2)	173(2)	298(2)	298(2)	173(2)	298(2)	173(2)	173(2)
λ (Å)	0.71073	0.71073	0.71073	0.71073	0.71073	0.71073	0.71073	0.71073	0.71073
Max. 2θ (°)	27.589	27.630	27.596	27.555	27.584	27.574	27.562	27.568	27.628
ρ_{calcd} (g cm^{-3})	4.406	4.560	4.570	4.543	4.568	4.640	4.618	4.480	4.510
μ (Mo K*α*) (mm^{-1})	36.472	35.649	35.930	35.767	36.183	36.886	36.908	34.204	34.701
GoF on F^2	1.058	1.060	1.055	1.026	1.013	1.071	1.028	1.057	1.054
R_1,[a] wR_2 [I > 2σ(I)][b]	0.0503, 0.1408	0.0453, 0.1065	0.0416, 0.1073	0.0580, 0.1112	0.0398 0.0816	0.0345, 0.0848	0.0417, 0.0903	0.0426, 0.0923	0.0524, 0.1230
R_1,[a] wR_2 (all data)[b]	0.0568, 0.1452	0.0696, 0.1156	0.0525, 0.1128	0.1207, 0.1298	0.0655, 0.0900	0.0415, 0.0875	0.0716, 0.0998	0.0612, 0.0983	0.0704, 0.1305
$(\Delta\rho)_{max}$, $(\Delta\rho)_{min}$/e·$Å^{-3}$	4.807, -6.447	5.088, -4.269	3.428, -5.164	2.818, -3.113	5.630, -2.278	3.922, -3.473	4.044, -2.840	3.755, -3.330	4.357, -5.930

Table S2. Comparison of sorption capacities and distribution coefficients of Re(VII)/Tc(VII) onto inorganic sorbents.

Sorbents	Experimental conditions	Adsorption capacity (mg/g)	K_d(mL/g)	reference
Biochar	T=298K; 12h; 3g/L	46.5	--	[4]
Nano-SiO_2	T=298K; pH2	4.94	--	[5]
Ni-Al LDH	Tc(VII); pH=7.9; 24h	--	1.5×10^3	[6]
PtGeS	Tc(VII); Ambient temperature; pH=4.8; 7 days	--	3.6×10^4	[7]
Mg- Al LDH	Ambient temperature; pH=7.0±0.1; 0.5g/L	130	122-300	[8]
NDTB-1	Tc(VII); Molar ratio TcO_4^-:NDTB-1=1:32	162	1.1×10^4	[9]
$Y_2(OH)_5Cl$	Ambient temperature; pH=7.0±0.1; 0.5g/L	27.5	71-144	[10]
$Yb_3O(OH)_3Cl$	Ambient temperature; pH=7.0±0.1; 0.5g/L	48.6	98-204	[10]
**$YPbOClO_4$-1**	**Ambient temperature; pH=6.0±0.1; 1g/L**	**434.7**	**3.8×10^3**	**Present work**
**$ErPbOClO_4$-2**	**Ambient temperature; pH=8; 1g/L**	**427.7**	**1.4×10^4**	**Present work**

Table S3. Crystallographic data for **$ErPbOReO_4$**.

Compound	**$ErPbOReO_4$**
Formula	$Er_6Pb_{18}Re_{12.25}O_{90.5}$
Mass	8461.93
Color	Purple
Habit	Tablet
Space Group	$P\bar{1}$
a (Å)	15.242(5)
b (Å)	15.956(6)
c (Å)	20.588(8)
α (°)	95.370(10)
β (°)	91.270(9)
γ (°)	93.579(10)
V (Å^3)	4974(3)
Z	2
T (K)	173(2)
λ (Å)	0.71073
Max. 2θ (°)	27.656
ρ_{calcd} (g cm^{-3})	5.650
μ (Mo Kα) (mm^{-1})	50.268
GoF on F^2	1.017
R_1,[a] wR_2 [I > 2σ(I)][b]	0.0720, 0.1260
R_1,[a] wR_2 (all data)[b]	0.1567, 0.1519
$(\Delta\rho)_{max}$, $(\Delta\rho)_{min}$/e·Å^{-3}	5.362, -5.151

Table S4. Pb-O and $Pb^{2+}\cdots O\text{-}ReO_3^-$ bond distances of compound **ErPbOReO$_4$**.

Bond type	Bond	Distances (Å)	Bond	Distances (Å)
Pb-O	Pb(1)-O(24)	2.254(18)	Pb(3)-O(25)	2.304(17)
	Pb(1)-O(33)	2.298(17)	Pb(3)-O(35)	2.329(19)
	Pb(1)-O(23)#1	2.346(18)	Pb(3)-O(27)	2.346(17)
$Pb^{2+}\cdots O\text{-}ReO_3^-$	Pb(1)-O(45)	2.73(2)		
Pb-O	Pb(2)-O(28)	2.286(18)	Pb(6)-O(33)	2.226(17)
	Pb(2)-O(34)	2.316(19)	Pb(6)-O(26)	2.300(17)
	Pb(2)-O(25)	2.386(18)	Pb(6)-O(27)	2.369(17)
$Pb^{2+}\cdots O\text{-}ReO_3^-$	Pb(2)-O(49)	2.75(2)		
Pb-O	Pb(4)-O(10)	2.322(17)	Pb(9)-O(7)	2.346(18)
	Pb(4)-O(36)	2.350(19)	Pb(9)-O(34)	2.35(2)
	Pb(4)-O(15)	2.360(17)	Pb(9)-O(3)#1	2.356(18)
$Pb^{2+}\cdots O\text{-}ReO_3^-$	Pb(4)-O(69)	2.71(2)		
Pb-O	Pb(5)-O(37)	2.292(17)	Pb(10)-O(21)	2.263(18)
	Pb(5)-O(23)	2.333(18)	Pb(10)-O(35)	2.300(18)
	Pb(5)-O(22)	2.362(17)	Pb(10)-O(22)	2.330(17)
$Pb^{2+}\cdots O\text{-}ReO_3^-$	Pb(5)-O(46)#3	2.69(2)		
Pb-O	Pb(7)-O(18)	2.283(17)	Pb(11)-O(38)	2.27(2)
	Pb(7)-O(36)	2.304(18)	Pb(11)-O(12)	2.280(18)
	Pb(7)-O(17)	2.369(18)	Pb(11)-O(15)	2.335(18)
$Pb^{2+}\cdots O\text{-}ReO_3^-$	Pb(7)-O(53)	2.70(2)		

Pb-O	Pb(8)-O(17)	2.328(18)	Pb(13)-O(38)	2.245(17)
	Pb(8)-O(39)	2.335(18)	Pb(13)-O(19)#2	2.30(2)
	Pb(8)-O(20)#2	2.389(18)	Pb(13)-O(20)	2.314(18)
$Pb^{2+}\cdots O\text{-}ReO_3^-$	Pb(8)-O(54)	2.75(2)		
Pb-O	Pb(12)-O(33)	2.212(18)	Pb(15)-O(31)	2.28(2)
	Pb(12)-O(6)	2.321(18)	Pb(15)-O(32)	2.33(2)
	Pb(12)-O(7)#1	2.411(17)	Pb(15)-O(39)	2.339(19)
$Pb^{2+}\cdots O\text{-}ReO_3^-$	Pb(12)-O(73)	2.73(2)		
Pb-O	Pb(14)-O(8)	2.235(18)	Pb(17)-O(32)	2.329(19)
	Pb(14)-O(3)	2.339(18)	Pb(17)-O(40)	2.34(2)
	Pb(14)-O(37)	2.350(18)	Pb(17)-O(30)	2.368(19)
$Pb^{2+}\cdots O\text{-}ReO_3^-$	Pb(14)-O(72)#4	2.73(4)		
Pb-O	Pb(16)-O(38)	2.21(2)	Pb(18)-O(14)	2.213(18)
	Pb(16)-O(29)	2.260(17)	Pb(18)-O(40)#2	2.32(2)
	Pb(16)-O(30)	2.345(19)	Pb(18)-O(10)	2.372(18)
$Pb^{2+}\cdots O\text{-}ReO_3^-$	Pb(16)-O(42)	2.74(2)		

Table S5. Selected bond distances and BVS of Y and Pb atoms for **$YPbOClO_4$-1**.

Bond	Distance (Å)	Bond	Distance (Å)
Pb(1)-O(6)	2.320(11)	Y(1)-O(3)	2.337(9)
Pb(1)-O(12)	2.326(10)	Y(1)-O(5)	2.346(9)
Pb(1)-O(18)	2.365(11)	Y(1)-O(2)#1	2.355(9)
Pb(1) BVS	1.635	Y(1)-O(1)#1	2.356(9)
Pb(2)-O(4)	2.282(10)	Y(1)-O(17)#1	2.361(10)
Pb(2)-O(18)	2.311(11)	Y(1)-O(4)	2.369(10)
Pb(2)-O(9)	2.359(9)	Y(1)-O(9)	2.417(10)
Pb(2) BVS	1.729	Y(1)-O(10)#1	2.424(10)
Pb(3)-O(15)	2.313(11)	Y(1) BVS	3.061
Pb(3)-O(10)	2.342(10)	Y(2)-O(1)	2.336(9)
Pb(3)-O(9)#1	2.347(10)	Y(2)-O(8)	2.341(10)
Pb(3) BVS	1.648	Y(2)-O(3)#1	2.348(9)
Pb(4)-O(11)	2.242(10)	Y(2)-O(5)	2.350(9)
Pb(4)-O(15)	2.293(11)	Y(2)-O(2)#1	2.364(9)
Pb(4)-O(7)	2.357(10)	Y(2)-O(16)	2.380(10)
Pb(4) BVS	1.833	Y(2)-O(12)	2.420(9)
Pb(5)-O(8)	2.243(10)	Y(2)-O(6)	2.426(11)
Pb(5)-O(13)	2.307(11)	Y(2) BVS	3.063
Pb(5)-O(12)	2.399(10)	Y(3)-O(3)	2.331(9)
Pb(5) BVS	1.753	Y(3)-O(2)	2.337(9)
Pb(6)-O(13)	2.318(11)	Y(3)-O(1)	2.339(10)
Pb(6)-O(7)	2.333(10)	Y(3)-O(5)	2.352(10)
Pb(6)-O(14)	2.367(10)	Y(3)-O(11)	2.356(10)
Pb(6) BVS	1.625	Y(3)-O(20)	2.369(10)
Pb(7)-O(19)	2.255(13)	Y(3)-O(14)	2.431(10)
Pb(7)-O(16)	2.293(11)	Y(3)-O(7)	2.440(10)
Pb(7)-O(6)	2.369(11)	Y(3) BVS	3.079
Pb(7) BVS	1.792		
Pb(8)-O(19)	2.223(14)		
Pb(8)-O(17)	2.265(11)		
Pb(8)-O(10)	2.380(10)		
Pb(8) BVS	1.887		
Pb(9)-O(19)#1	2.275(14)		
Pb(9)-O(20)	2.308(11)		
Pb(9)-O(14)	2.369(11)		
Pb(9) BVS	1.732		

Table S6. Selected bond distances and BVS of Er and Pb atoms for **$ErPbOClO_4$-2**.

Bond	Distance	Bond	Distance	Bond	Distance	Bond	Distance

	(Å)		(Å)
Pb(1)-O(27)	2.256 (9)	Pb(9)-O(26)	2.276 (9)
Pb(1)-O(30)	2.271 (9)	Pb(9)-O(32)	2.335 (9)
Pb(1)-O(37)	2.358 (9)	Pb(9)-O(25)	2.344 (10)
Pb(1) BVS	1.840	Pb(9) BVS	1.729
Pb(2)-O(28)	2.256 (9)	Pb(10)-O(11)	2.328 (9)
Pb(2)-O(12)	2.299 (10)	Pb(10)-O(24)	2.331 (9)
Pb(2)-O(33)	2.323 (9)	Pb(10)-O(12)	2.361 (10)
Pb(2) BVS	1.846	Pb(10) BVS	1.650
Pb(3)-O(20)	2.218 (9)	Pb(11)-O(14)	2.285 (9)
Pb(3)-O(17)	2.270 (10)	Pb(11)-O(38)	2.373 (10)
Pb(3)-O(22)	2.308 (9)	Pb(11)-O(11)	2.377 (9)
Pb(3) BVS	1.937	Pb(11) BVS	1.658
Pb(4)-O(39)	2.231 (9)	Pb(12)-O(29)	2.283 (9)
Pb(4)-O(36)	2.300 (10)	Pb(12)-O(22)	2.299 (9)
Pb(4)-O(32)	2.349 (9)	Pb(12)-O(36)	2.377 (10)
Pb(4) BVS	1.826	Pb(12) BVS	1.743
Pb(5)-O(21)	2.269 (10)	Pb(13)-O(9)	2.274 (9)
Pb(5)-O(25)#2	2.268 (9)	Pb(13)-O(23)	2.304 (9)
Pb(5)-O(10)	2.367 (9)	Pb(13)-O(15)	2.305 (9)
Pb(5) BVS	1.830	Pb(13) BVS	1.760
Pb(6)-O(33)#1	2.319 (10)	Pb(14)-O(19)	2.276 (9)
Pb(6)-O(23)	2.345 (9)	Pb(14)-O(17)	2.303 (10)
Pb(6)-O(18)	2.373 (9)	Pb(14)-O(10)	2.359 (9)
Pb(6) BVS	1.582	Pb(14) BVS	1.819
Pb(7)-O(27)#1	2.270 (9)	Pb(15)-O(27)#1	2.279(9)
Pb(7)-O(16)	2.289 (9)	Pb(15)-O(35)#1	2.298(9)
Pb(7)-O(24)	2.399 (9)	Pb(15)-O(18)	2.364(9)
Pb(7) BVS	1.751	Pb(15) BVS	1.745
Pb(8)-O(38)	2.274 (9)		
Pb(8)-O(15)	2.288 (9)		
Pb(8)-O(37)	2.327 (9)		

	(Å)		(Å)
Er(1)-O(8)#1	2.323(8)	Er(4)-O(5)	2.337(9)
Er(1)-O(7)	2.335(8)	Er(4)-O(29)	2.346(9)
Er(1)-O(2)	2.339(8)	Er(4)-O(20)	2.355(9)
Er(1)-O(4)	2.340(8)	Er(4)-O(1)	2.356(9)
Er(1)-O(35)	2.350(8)	Er(4)-O(6)	2.361(10)
Er(1)-O(28)	2.351(9)	Er(4)-O(3)	2.369(10)
Er(1)-O(18)#1	2.420(8)	Er(4)-O(31)	2.417(10)
Er(1)-O(33)	2.468(9)	Er(4)-O(22)	2.424(10)
Er(1) BVS	3.082	Er(4) BVS	3.103
Er(2)-O(3)	2.331(8)	Er(5)-O(2)#1	2.336(9)
Er(2)-O(26)	2.338(8)	Er(5)-O(7)	2.341(10)
Er(2)-O(5)	2.342(7)	Er(5)-O(4)#1	2.348(9)
Er(2)-O(6)#2	2.344(8)	Er(5)-O(16)	2.350(9)
Er(2)-O(1)#2	2.346(8)	Er(5)-O(8)#1	2.364(9)
Er(2)-O(39)	2.355(8)	Er(5)-O(14)	2.380(10)
Er(2)-O(13)	2.382(9)	Er(5)-O(24)	2.420(9)
Er(2)-O(32)	2.448(8)	Er(5)-O(11)	2.426(11)
Er(2) BVS	3.114	Er(5) BVS	3.055
Er(3)-O(19)	2.317(8)	Er(6)-O(2)	2.328(8)
Er(3)-O(21)	2.323(8)	Er(6)-O(8)	2.329(8)
Er(3)-O(1)	2.326(8)	Er(6)-O(4)#1	2.334(8)
Er(3)-O(5)	2.337(7)	Er(6)-O(30)	2.354(8)
Er(3)-O(3)#2	2.348(8)	Er(6)-O(7)	2.358(8)
Er(3)-O(6)#2	2.352(8)	Er(6)-O(9)	2.376(8)
Er(3)-O(34)	2.401(8)	Er(6)-O(37)	2.406(8)
Er(3)-O(10)	2.477(8)	Er(6)-O(15)	2.445(8)
Er(3) BVS	3.132	Er(6) BVS	3.071

Pb(8) BVS	1.793	

Table S7. Selected bond distances and BVS of O atoms for **YPbOClO4-1**.

Bond	Distance (Å)	Bond	Distance (Å)	Bond	Distance (Å)
O(1)#1-Y(1)	2.356(9)	O(8)-Pb(5)	2.243(10)	O(15)-Pb(3)	2.313(11)
O(1)-Y(2)	2.336(9)	O(8)-Y(2)	2.341(10)	O(15)-Pb(4)	2.293(11)
O(1)-Y(3)	2.339(10)				
O(1) BVS	1.231	O(8) BVS	0.952	O(15) BVS	0.916
O(2)#1-Y(1)	2.355(9)	O(9)-Pb(2)	2.359(9)	O(16)-Pb(7)	2.293(11)
O(2)#1-Y(2)	2.364(9)	O(9)#1-Pb(3)	2.347(10)	O(16)-Y(2)	2.380(10)
O(2)-Y(3)	2.337(9)	O(9)-Y(1)	2.417(10)		
O(2) BVS	1.204	O(9) BVS	1.137	O(16) BVS	0.842
O(3)-Y(1)	2.337(9)	O(10)-Pb(3)	2.342(10)	O(17)-Pb(8)	2.265(11)
O(3)#1-Y(2)	2.348(9)	O(10)-Pb(8)	2.380(10)	O(17)#1-Y(1)	2.361(10)
O(3)-Y(3)	2.331(9)	O(10)#1-Y(1)	2.424(10)		
O(3) BVS	1.248	O(10) BVS	1.114	O(17) BVS	0.899
O(4)-Y(1)	2.369(10)	O(11)-Pb(4)	2.242(10)	O(18)-Pb(1)	2.365(11)
O(4)-Pb(2)	2.282(10)	O(11)-Y(3)	2.356(10)	O(18)-Pb(2)	2.311(11)
O(4) BVS	0.868	O(11) BVS	0.937	O(18) BVS	0.835
O(5)-Y(1)	2.346(9)	O(12)-Pb(1)	2.326(10)	O(19)-Pb(7)	2.255(13)
O(5)-Y(2)	2.350(9)	O(12)-Pb(5)	2.399(10)	O(19)-Pb(8)	2.223(14)
O(5)-Y(3)	2.352(10)	O(12)-Y(2)	2.420(9)	O(19)#1-Pb(9)	2.275(14)
O(5) BVS	1.212	O(12) BVS	1.117	O(19) BVS	1.584
O(6)-Y(2)	2.426(11)	O(13)-Pb(5)	2.307(11)	O(20)-Pb(9)	2.308(11)
O(6)-Pb(7)	2.369(11)	O(13)-Pb(6)	2.318(11)	O(20)-Y(3)	2.369(10)
O(6)-Pb(1)	2.320(11)				
O(6) BVS	1.149	O(13) BVS	0.893	O(20) BVS	0.834
O(7)-Pb(4)	2.357(10)	O(14)-Pb(6)	2.367(10)		
O(7)-Pb(6)	2.333(10)	O(14)-Pb(9)	2.369(11)		
O(7)-Y(3)	2.440(10)	O(14)-Y(3)	2.431(10)		
O(7) BVS	1.134	O(14) BVS	1.092		

Table S8. Selected bond distances and BVS of O atoms for **ErPbOClO$_4$-2**.

Bond	Distance (Å)	Bond	Distance (Å)	Bond	Distance (Å)
O(1)#2-Er(2)	2.346(8)	O(15)- Er(6)	2.445(8)	O(29)- Er(4)	2.325(8)
O(1)-Er(3)	2.326(8)	O(15)- Pb(8)	2.293(8)	O(29)- Pb(12)	2.280(9)
O(1)-Er(4)	2.337(8)	O(15)- Pb(13)	2.397(8)		
O(1) BVS	1.242	O(15) BVS	1.385	O(29) BVS	1.062
O(2)#1-Er(5)	2.321(7)	O(16)- Er(5)	2.345(8)	O(30)- Er(6)	2.354(8)
O(2)-Er(6)	2.328(8)	O(16)- Pb(7)	2.278(9)	O(30)- Pb(1)	2.278(8)
O(2)-Er(1)	2.339(8)				
O(2) BVS	1.266	O(16) BVS	1.043	O(30) BVS	1.033
O(3)-Er(2)	2.331(8)	O(17)- Pb(3)	2.271(9)	O(31)- Er(4)	2.412(9)
O(3)#2-Er(3)	2.348(8)	O(17)- Pb(14)	2.310(9)		
O(3)-Er(4)	2.351(8)				
O(3) BVS	1.220	O(17) BVS	1.236	O(31) BVS	0.337
O(4)#1-Er(5)	2.343(8)	O(18)#1- Er(1)	2.420(8)	O(32)- Er(2)	2.448(8)
O(4)#1-Er(6)	2.334(8)	O(18)- Pb(6)	2.374(8)	O(32)- Pb(4)	2.357(9)
O(4)- Er(1)	2.340(8)	O(18)- Pb(15)	2.364(9)	O(32)- Pb(9)	2.332(8)
O(4) BVS	1.233	O(18) BVS	1.329	O(32) BVS	1.374
O(5)-Er(3)	2.337(7)	O(19)- Er(3)	2.317(8)	O(33)- Er(1)-	2.468(9)
O(5)-Er(4)	2.324(7)	O(19)- Pb(14)	2.269(8)	O(33)- Pb(2)	2.329(9)
O(5)-Er(2)	2.342(7)			O(33)#1- Pb(6)	2.334(8)
O(5) BVS	1.249	O(19) BVS	1.090	O(33) BVS	1.395
O(6)#2-Er(3)	2.352(8)	O(20)- Er(4)	2.333(8)	O(34)- Er(3)	2.401(8)
O(6)-Er(4)	2.350(8)	O(20)- Pb(3)	2.238(8)		
O(6)#2-Er(2)	2.344(8)				
O(6) BVS	1.201	O(20) BVS	1.129	O(34) -BVS	0.348
O(7)-Er(5)	2.337(8)	O(21)- Er(3)	2.323(8)	O(35)-Er(1)	2.350(8)
O(7)-Er(6)	2.358(8)	O(21)- Pb(5)	2.248(9)	O(35)#1-Pb(15)	2.298(9)
O(7)-Er(1)	2.335(8)				
O(7) BVS	1.219	O(21) BVS	1.122	O(35) BVS	1.004
O(8)#1-Er(5)	2.373(8)	O(22)- Er(4)	2.477(8)	O(36)-Pb(4)	2.289(9)
O(8)-Er(6)	2.329(8)	O(22)- Pb(3)	2.317(8)	O(36)-Pb(12)	2.372(9)
O(8)#1-Er(1)	2.323(8)	O(22)- Pb(12)	2.293(8)		
O(8) BVS	1.411	O(22) BVS	1.471	O(36) BVS	1.115
O(9)-Er(6)	2.376(8)	O(23)- Pb(6)	2.339(9)	O(37)-Er(6)	2.406(8)
O(9)-Pb(13)	2.255(8)	O(23)- Pb(13)	2.290(9)	O(37)-Pb(1)	2.354(8)
				O(37)-Pb(8)	2.332(8)
O(9) BVS	1.051	O(23) BVS	1.160	O(37) BVS	1.415
O(10)-Pb(5)	2.366(8)	O(24)-Er(5)	2.408(8)	O(38)- Pb(8)	2.284(9)
O(10)-Pb(14)	2.314(8)	O(24)-Pb(7)	2.398(8)	O(38)-Pb(11)	2.352(9)
O(10)-Er(3)	2.477(8)	O(24)-Pb(10)	2.333(8)		
O(10) BVS	1.366	O(24) BVS	1.353	O(38) BVS	1.151
O(11)-Er(5)	2.431(8)	O(25)#2-Pb(5)	2.280(9)	O(39)-Er(2)	2.355(8)
O(11)-Pb(10)	2.325(8)	O(25)-Pb(9)	2.336(9)	O(39)-Pb(4)	2.249(8)
O(11)-Pb(11)-	2.376(9)				
O(11) BVS	1.373	O(25) BVS	1.181	O(39) BVS	1.084

O(12)-Pb(2)	2.305(9)	O(26)-Er(2)	2.338(8)		
O(12)-Pb(10)	2.342(9)	O(26)-Pb(9)	2.282(8)		
O(12) BVS	1.131	O(26) BVS	1.044		
O(13)-Er(2)	2.382(9)	O(27)-Pb(1)	2.254(9)		
		O(27)#1-Pb(7)	2.271(9)		
		O(27)#1-Pb(15)	2.279(9)		
O(13) BVS	0.366	O(27) BVS	1.969		
O(14)-Er(5)	2.385(8)	O(28)-Er(1)	2.351(9)		
O(14)-Pb(11)	2.274(9)	O(28)-Pb(2)	2.246(9)		
O(14) BVS	1.008	O(28) BVS	1.094		

Table S9. Fitting results based on the pseudo-first-order and pseudo-second-order kinetics models.

Samples	Kinetics model					
	Pseudo-first-order			Pseudo-second-order		
	q_e(mg/g)	k_1(min^{-1})	R^2	q_e(mg/g))	k_2(g mg^{-1} min^{-1}))	R^2
**$YPbOClO_4$-1**	26.5	5.3×10^{-3}	0.86	31.5	7.8×10^{-4}	＞0.99
**$ErPbOClO_4$-2**	19.8	6.2×10^{-3}	0.97	37.9	9.6×10^{-4}	＞0.99

Table S10. The ReO_4^- removal percentage in the presence of different anions by **$YPbOClO_4$-1** and **$ErPbOClO_4$-2**.

Solution	$YPbOClO_4$-1		$ErPbOClO_4$-2	
	ReO_4^- (ppm)	% ReO_4^- removal	ReO_4^- (ppm)	% ReO_4^- removal
**ReO_4^- + Cl^- Stock**	95.2	--	95.2	--
**After ion-exchange**	6.0	91.9%	6.9	91.8%
**ReO_4^- + CO_3^{2-} Stock**	94.5	--	94.5	--
**After ion-exchange**	4.8	94.9%	7.9	90.4%
**ReO_4^- + $B(OH)_4^-$ Stock**	97.1	--	97.1	--
**After ion-exchange**	6.7	91.1%	7.0	92.6%
**ReO_4^- + PO_4^{3-} Stock**	90.5	--	90.5	--
**After ion-exchange**	4.8	93.0%	9.1	89.4%

**ReO_4^- + SO_4^{2-} Stock**	95.8	--	95.8	--
**After ion-exchange**	11.9	86.0%	19.5	77.8%

S3. REFERENCE

[1] L. Atanasoska, K. Naoi, W. H. Smyrl, *Chem. Mater.* **1992**, *4*, 988-994.

[2] J. F. Moulder, J. Chastain, R. C. King, *Handbook of X-ray Photoelectron Spectroscopy: A Reference Book of Standard Spectra for Identification and Interpretation of XPS Data*, Physical Electronics, **1995**.

[3] V. Bondarenka, S. Grebinskij, S. Kaciulis, G. Mattogno, S. Mickevicius, H. Tvardauskas, V. Volkov, G. Zakharova, *J. Electron. Spectrosc. Relat. Phenom.* **2001**, *120*, 131-135.

[4] J.-H. Zu, Y.-Z. Wei, M.-S. Ye, F.-D. Tang, L.-F. He, R.-Q. Liu, *Nucl. Sci. Tech.* **2015**, *26*, 69-75.

[5] Y. Li, Q. Wang, Q. Li, Z. Zhang, L. Zhang, X. Liu, *J. Taiwan Inst. Chem. E.* **2015**, *55*, 126-132.

[6] K.-H. Goh, T.-T. Lim, Z. Dong, *Water Res.* **2008**, *42*, 1343-1368.

[7] B. J. Riley, J. Chun, W. Um, W. C. Lepry, J. Matyas, M. J. Olszta, X. Li, K. Polychronopoulou, M. G. Kanatzidis, *Environ. Sci. Technol.* **2013**, *47*, 7540-7547.

[8] L. Zhu, L. Zhang, J. Li, D. Zhang, L. Chen, D. Sheng, S. Yang, C. Xiao, J. Wang, Z. Chai, T. E. Albrecht-Schmitt, S. Wang, *Environ. Sci. Technol.* **2017**, *51*, 8606-8615.
[9] S. Wang, P. Yu, B. A. Purse, M. J. Orta, J. Diwu, W. H. Casey, B. L. Phillips, E. V. Alekseev, W. Depmeier, D. T. Hobbs, T. E. Albrecht-Schmitt, *Adv. Funct. Mater.* **2012**, *22*, 2241-2250.
[10] D. Sheng, L. Zhu, C. Xu, C. Xiao, Y. Wang, Y. Wang, L. Chen, J. Diwu, J. Chen, Z. Chai, T. E. Albrecht-Schmitt, S. Wang, *Environ. Sci. Technol.* **2017**, *55*, 3721–3723.
